# ALK signaling cascade confers multiple advantages to glioblastoma cells through neovascularization and cell proliferation

**DOI:** 10.1371/journal.pone.0183516

**Published:** 2017-08-24

**Authors:** Risako Chiba, Masashi Akiya, Miki Hashimura, Yasuko Oguri, Madoka Inukai, Atsuko Hara, Makoto Saegusa

**Affiliations:** Department of Pathology, Kitasato University School of Medicine, Minami-ku, Sagamihara, Kanagawa, Japan; Kyung Hee University, REPUBLIC OF KOREA

## Abstract

Anaplastic lymphoma kinase (ALK), which is a receptor tyrosine kinase, is essentially and transiently expressed in the developing nervous system. Here we examined the functional role of the *ALK* gene in glioblastomas (GBMs). In clinical samples of GBMs, high ALK expression without gene rearrangements or mutations was frequently observed in perivascular lesions, in contrast to the relatively low expression in the perinecrotic areas, which was positively correlated with N-myc and phosphorylated (p) Stat3 scores and Ki-67 labeling indices. ALK immunoreactivity was also found to be associated with neovascular features including vascular co-option and vascular mimicry. In astrocytoma cell lines, cells stably overexpressing full-length ALK showed an increase in expression of pStat3 and pAkt proteins, as well as hypoxia-inducible factor-1α (HIF-1α) and vascular endothelial growth factor-A (VEGF-A) mRNAs, in contrast to cells with knockdown of endogenous ALK which showed decreased expression of these molecules. Transfection of the constitutively active form of Stat3 induced an increase in *HIF-1*α promoter activity, and the overexpression of HIF-1α in turn resulted in enhancement of *VEGF-A* promoter activity. In addition, cells with overexpression or knockdown of ALK also showed a tendency toward increased and decreased proliferation, respectively, through changes in expression of pAkt and pStat3. Finally, *ALK* promoter was significantly activated by transfection of Sox4 and N-myc, which are known to contribute to neuronal properties. These findings therefore suggest that N-myc/Sox4-mediated ALK signaling cascades containing Stat3, Akt, HIF-1α, and VEGF-A confer multiple advantages to tumor growth through alterations in neovascularization and cell proliferation in GBMs.

## Introduction

Glioblastoma (GBM), also referred to as World Health Organization grade IV astrocytoma, is one of the most vascular-rich tumors and is characterized by vascular proliferation in response to abundant vascular endothelial growth factor (VEGF) which is produced by tumor cells [1–3]. The tumor vessels are morphologically and functionally different from normal blood vessels, and at least five distinct mechanisms of neovascularization in GBM have been identified: i) vascular co-option, ii) angiogenesis, iii) vasculogenesis, iv) vascular mimicry, and v) glioblastoma-endothelial cell trans-differentiation. These mechanisms are not independent of one another, but rather are interlinked and are controlled, at least in part, by similar processes [4–7].

An increasing body of evidence suggests that hypoxia plays an important role in creating a microenvironment that provides the essential cellular interaction through activation of hypoxia-inducible factor (HIF) signaling and induction of VEGF expression in hypoxia-sensing cells [8,9]. Thus, activation of HIF-1 pathway is a common feature of glioma and may explain the intense vascular hyperplasia often seen in GBMs. Recently, there is also evidence demonstrating the existence of hypoxia-independent VEGF-mediated pathways [10–12].

The *anaplastic lymphoma kinase (ALK)* gene, located on chromosome 2p23, is highly homologous to that of *leukocyte tyrosine kinase* and further belongs to the insulin receptor superfamily of receptor tyrosine kinases [13–17]. Full-length ALK is specifically expressed in the developing central and peripheral nervous systems during embryogenesis, and is associated with the balance of cell proliferation and differentiation [18–20]. While several fusion genes involving *ALK* produced by chromosomal rearrangements have been found in a subset of lymphomas and lung carcinomas [14,21]. recently, deregulated expression of full-length ALK has also been observed in some primary solid tumors derived from various tissues, including GBMs and neuroblastomas [22]. In addition, constitutively activated ALK causes increased VEGF secretion in anaplastic large-cell lymphoma [23].

In implanted murine gliomas with production of pleiotrophin that acts as an upstream regulator of ALK signaling, treatment with an ALK inhibitor reduced vascular density and decreased vascular diameter [24]. Together with the evidence of upregulation of HIF-1α mRNA in ALK-positive anaplastic large cell lymphoma under normoxic condition [25], we therefore hypothesized that ALK signaling may contribute to tumor growth through hypoxia-independent neovascularization in GBMs. To test this, we hereby investigated the expression of ALK, as well as the profiles of its related molecules, using clinical samples and cell lines of GBMs.

## Materials and methods

### Clinical cases

A total of 141 cases of astrocytomas, surgically resected at the Kitasato University hospital in the period from 1995 to 2013, were selected from our patient records according to the criteria of the 2015 WHO classification [26]. The mean age of the patients was 54.3 years (range, 1 to 79 years). Of these, 53, 36, and 52 cases were subcategorized as GII, GIII, and GIV (GBM), respectively. None of the patients were treated with chemo-radiation therapy before surgical resection of the tumors. In GBM cases, tumor areas were subdivided into two categories, including perivascular components and perinecrotic areas with or without pseudopalisading features. All tissues were routinely fixed in 10% formalin and processed for embedment in paraffin wax (FFPE). Approval for this study was given by the Ethics Committee of the Kitasato University School of Medicine (B15-213).

### Antibodies and reagent

Anti-ALK antibody (5A4), as well as ALK iAEP kit, were obtained from Nichirei Biosciences (Tokyo, Japan). Anti-ALK (D5F3), anti-phospho(p)-Akt at serine (Ser) 473 (pAkt), anti-Akt, anti-pStat3 (Tyr705)(D3A7), and anti-Stat3 (79D7) antibodies were purchased from Cell Signaling (Danvers, MA, USA). Anti-N-myc and anti-CD34 (rabbit) antibodies were obtained from Abcam (Cambridge, MA, USA). Anti-c-myc antibody was from Santa Cruz Biotechnology (Santa Cruz, CA, USA). Anti-HIF-1α and anti-IDH1 R132H antibodies were from BD Biosciences (San Jose, CA, USA) and Dianova GmbH (Hamburg, Germany), respectively. Anti-CD34 (mouse), anti-human smooth muscle actin (SMA), and anti-Ki-67 antibodies were purchased from Dako (Glostrup, Denmark). Anti-β-actin antibody and cobalt chloride (CoCl_2_) were from Sigma-Aldrich Chemicals (St. Louis, MO, USA).

### Immunohistochemistry (IHC)

IHC was performed using a combination of microwave-oven heating and Histofine Simple Stain MAX-PO (MULTI) (Nichirei Biosciences) methods. Lung carcinoma tissues with ALK overexpression due to the gene abnormality were used as positive control (S1 Fig). Both ALK/CD34 and SMA/CD34 double-stainings were also carried out using a combination of either ALK iAEP kit or Histofine Simple Stain MAX-PO (MULTI) and Histofine Simple Stain AP (R) (Nichirei Biosciences). In addition, CD34/periodic acid-Schiff (PAS) double-staining was applied for evaluation of vascular mimicry.

For evaluation of the IHC findings, scoring for ALK, N-myc, c-myc, pStat3, pAkt, and HIF-1α were carried out. Briefly, cases were subdivided into five categories on the basis of the proportion of immunopositive cells, as follows: 0, all negative; 1, <25%; 2, 25–50%; 3, 50–75%; and 4, >75% positive cells. The immunointensity was also subcategorized into four groups, as follows: 0, negative; 1, weak; 2, moderate; and 3, strong immunointensity. The IHC scores for each case were produced by multiplication of the values of the two parameters. To determine labeling indices (LIs) for Ki-67, the immunopositive nuclei of at least 700 tumor cells were counted in five randomly selected fields. The LIs were then calculated as number per 100 cells. In GBM cases, the IHC score and nuclear LI were also examined in perivasuclar and perinecrotic lesions separately. Immunopositivity for IDH1-R132H was defined as described previously [27].

### Evaluation of neovascularization features in GBMs

Neovascularization features including vascular co-option, which is characterized by the development of new vessels from pre-existing ones, and vascular mimicry, which refers to the formation of vessel-like structures from the lining of tumor cells, were evaluated in GBM tissues. Briefly, vascular co-option was defined as organization of GBM cells into cuffs around nonangiogenic microvessels as described by Holash et al [6]. To distinguish them from newly-formed vessels, the evaluation of vascular co-option was conducted in the tumor periphery adjacent to non-neoplastic brain tissues, since the vessel co-option components often are observed at actively growing edges, with the more mature center showing a switch to an angiogenic phenotype [28]. Vascular mimicry was also defined as CD34-negative and PAS-positive vascular channels (S2 Fig), as described previously [29].

### *In situ* hybridization (ISH)

Riboprobes for ALK containing nucleotides 3946 to 4633 of the *ALK* gene were generated by *in vitro* transcription, and ISH assays were performed using the GenPoint Tyramide Signal Amplification System (Dako) as described previously [27]. ISH signal was classified into four levels, as follows: -, none; 1+, fewer than 10% positive cells; 2+, 10–30%; and 3+, more than 30%. Samples with a score of either 1+, 2+, or 3+ were considered as positive and—was considered as negative.

### Fluorescence *in situ* hybridization (FISH)

For analysis of the *ALK* (2p23) locus, dual-color FISH studies were conducted on four GBM cases with strong ALK immunopositivity using the Vysis LSI ALK break-apart rearrangement probe (Abbott Molecular, Abbott Park, IL, USA), according to the manufacturer’s instructions. ProbeCheck ALK Positive Control Slides (Abbott Molecular) were also used as a positive control for *ALK* gene rearrangement.

### Mutation analyses of the *ALK* and *IDH1* genes

Genomic DNA was extracted from FFPE sections using the QIAamp DNA FFPE Tissue Kit (Qiagen, Valencia, CA, USA), according to the manufacturer’s instructions. Mutation analyses of exons 20, 23, 24, and 25 of the *ALK* gene and exon 4 of the *IDH1* gene were carried out as described previously [27,30].

### Plasmids and cell lines

Full-length cDNA of Stat3 (Open Biosystems, Huntsville, AL, USA) was cloned into pcDNA3.1 (Invitrogen, Carlsbad, CA, USA). The constitutively active mutant form of Stat3 (Stat3C) was further generated by site-directed mutagenesis to produce A662C and N664C using the PrimeSTAR Mutagenesis Basal kit (Takara Bio, Shiga, Japan) and a specific primer set (forward: 5’-TGGATTGTACCTGTATCCTGGTGTCTCCAC-3’ and reverse: 5’-AGGATACAGGTACAATCCATGATCTTATAGCC-3’). The human *VEGF-A* promoter (GenBank accession number NG_008732.1) between– 2000 and +50 bp (where +1 represents the transcription start site) was amplified by polymerase chain reaction (PCR) using a specific primer set (forward: 5’-ATAGGTACCAAGGAGGAAAGTTAGTGGCTTCCCTTCCAT-3’ and reverse: 5’AAACTCGAGCCCCCAGCGCCACGACCTCCGAGCT-3’), and was cloned into the pGL-3B vector (Promega, Madison, WT, USA). The pGL4-HIF-1α plasmid was obtained from the Riken DNA Bank Human Resource (Tsukuba, Japan). The pGL3B-ALK vector and its associated 5’-truncated promoter constructs, as well as those with site-directed mutagenesis in putative E1- and E2-boxes in the proximal promoter region, in addition to pGL3B-Sox4, pGL3B-N-myc, pcDNA3.1-full-length ALK, pcDNA3.1-c-myc, pcDNA3.1-Sox2, pcDNA3.1-Sox3, pcDNA3.1-Sox4, pcDNA-Sox5, pcDNA3.1-Sox6, pcDNA3.1-Sox7, pcDNA3.1-Sox9, pcDNA3.1-Sox11, pcDNA3.1-Sox17, and pSIREN-RetroQ-short hairpin (sh) ALK were also employed as described previously [31–33].

Three astrocytoma cell lines, KS-1 (GBM), KINGS-1 (anaplastic astrocytoma), and No.10 (anaplastic astrocytoma) were obtained from the Health Science Research Resources Bank (Osaka, Japan). The pcDNA3.1-full-length ALK or the pSIREN-RetroQ-shALK construct, with the associated empty vector as control, was transfected into KS-1 and KINGS-1 cells, respectively, and clones with ALK overexpression or knockdown were established as described previously [33]. Hec251 cells (endometrial cancer) stably overexpressing full-length ALK were used as a positive control for ALK expression [33].

### Reverse-transcription (RT)-PCR

cDNA was synthesized from 2 μg of total RNA. Amplification was carried out in the exponential phase to allow comparison among cDNAs synthesized from identical reactions using specific primers as described previously [31,34].

### Western blot assays

Total cellular proteins were isolated using RIPA buffer [20mM Tris-HCl (pH7.2), 1% Nonidet p-40, 0.5% sodium deoxycholate, 0.1% sodium dodecyl sulfate]. Western blot assay was carried out as described previously [31–33].

### Transfection

Transfection was carried out using LipofectAMINE PLUS (Invitrogen), in duplicate or triplicate as described previously [31–33]. Luciferase activity was assayed as described previously [31–33].

### Cell Counting Kit-8 assay

The quantitation of viable cells that reflect proliferation was carried out using a Cell Counting Kit-8 (CCK-8; Dojindo Lab, Kumamoto, Japan) according to the manufacturers’ instructions.

### Statistics

Comparative data were analyzed using the Mann-Whitney *U* test, chi-square test, and Spearman’s correlation coefficients. Overall survival (OS) was calculated as the time between onset and death or the date of the last follow-up evaluation. Progression-free survival (PFS) was also examined from the onset of treatment until relapse, disease progression, or last follow-up evaluation. OS and PSF were estimated using the Kaplan-Meier method, and statistical comparisons were made using the log rank test. The cut-off for statistical significance was set as p<0.05.

## Results

### ALK expression and its relation to prognosis in astrocytomas

**Fig 1:**
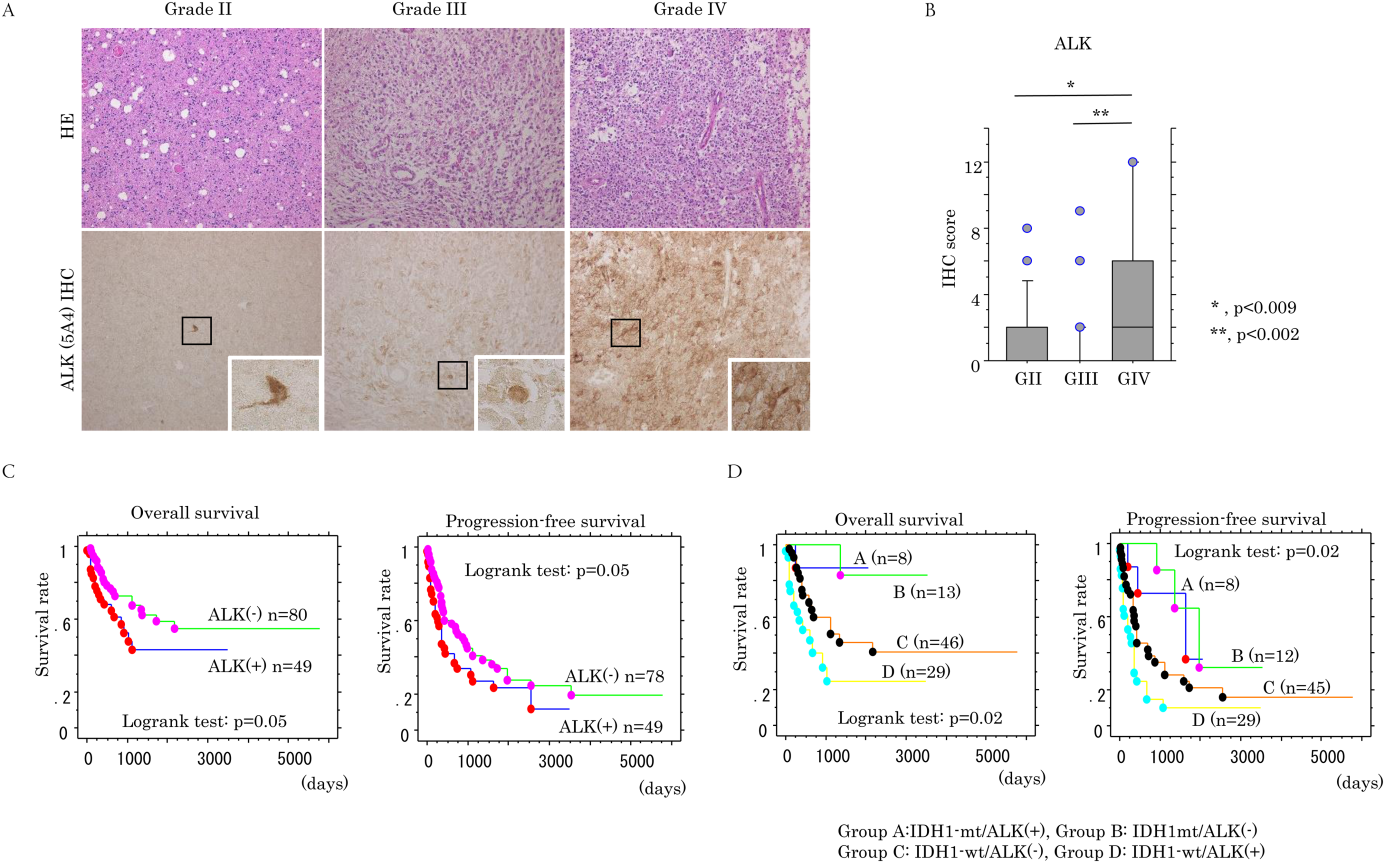
Up-regulation of ALK expression in GBMs. (A) Staining by hematoxylin and eosin (HE) and IHC for ALK in astrocytomas. Note the diffuse distribution of strong cytoplasmic ALK (5A4)-positive cells in grade IV (GBM) tumor, in contrast to the sporadic distribution or absence in grade II and III astrocytomas. Insets show the magnified views of the boxed area. Original magnification, x100 and x400 (inset). (B) IHC score for ALK (5A4) in astrocytomas. G, grade (C) Relationship of ALK (5A4) expression with overall survival and progression-free survival for all grades of astrocytomas. n, number of cases. (D) Relationship of ALK (5A4) expression and *isocitrate dehydrogenase 1 (IDH1)* gene status with overall survival and progression-free survival for all grades of astrocytomas. n, number of cases. wt, wild type; mt, mutant type.

Representative images of IHC findings for ALK detected by ALK iAEP kit containing anti-ALK antibody (5A4) in astrocytomas are illustrated in Fig 1A. Immunoreaction for ALK (5A4) was mainly observed in cytoplasmic components of astrocytoma cells. Similar findings were also observed when another antibody against ALK (D5F3) was applied, but the immunoreactivity in the latter was relatively weak as compared to that of the former (S3 Fig). In normal brain tissues, ALK (5A4) immunoreactivity was extremely low or absent in both astrocytes and nerve cells (S4 Fig).

ALK (5A4) immunopositivity (IHC score≧1) was evident in 9 (17.6%) of 51 GII, 5 (17.2%) of 29 GIII, and 29 (49.2%) of 52 GIV (GBM) astrocytomas, and the IHC scores were significantly higher in GBM as compared to those of GII and GIII tumors (Fig 1B).

The Kaplan-Meier curves for OS and PFS with respect to ALK (5A4) expression in astrocytomas showed that the patients with all grades of astrocytomas who displayed a lack of ALK (5A4) expression had more favorable OS and PFS as compared to the ALK (5A4)-immunopositive patients (Fig 1C). Although IDH1 alterations were significantly associated with favorable OS and PFS for all grades of astrocytomas (S5 Fig and S1 and S2 Tables), ALK (5A4) status did not affect the prognostic significance of *IDH1* gene alterations when the patients were subdivided into four groups on the basis of ALK (5A4) and IDH1 statuses (Fig 1D), in line with our previous study [25].

### Relationship between ALK expression and vascularization in GBMs

**Fig 2:**
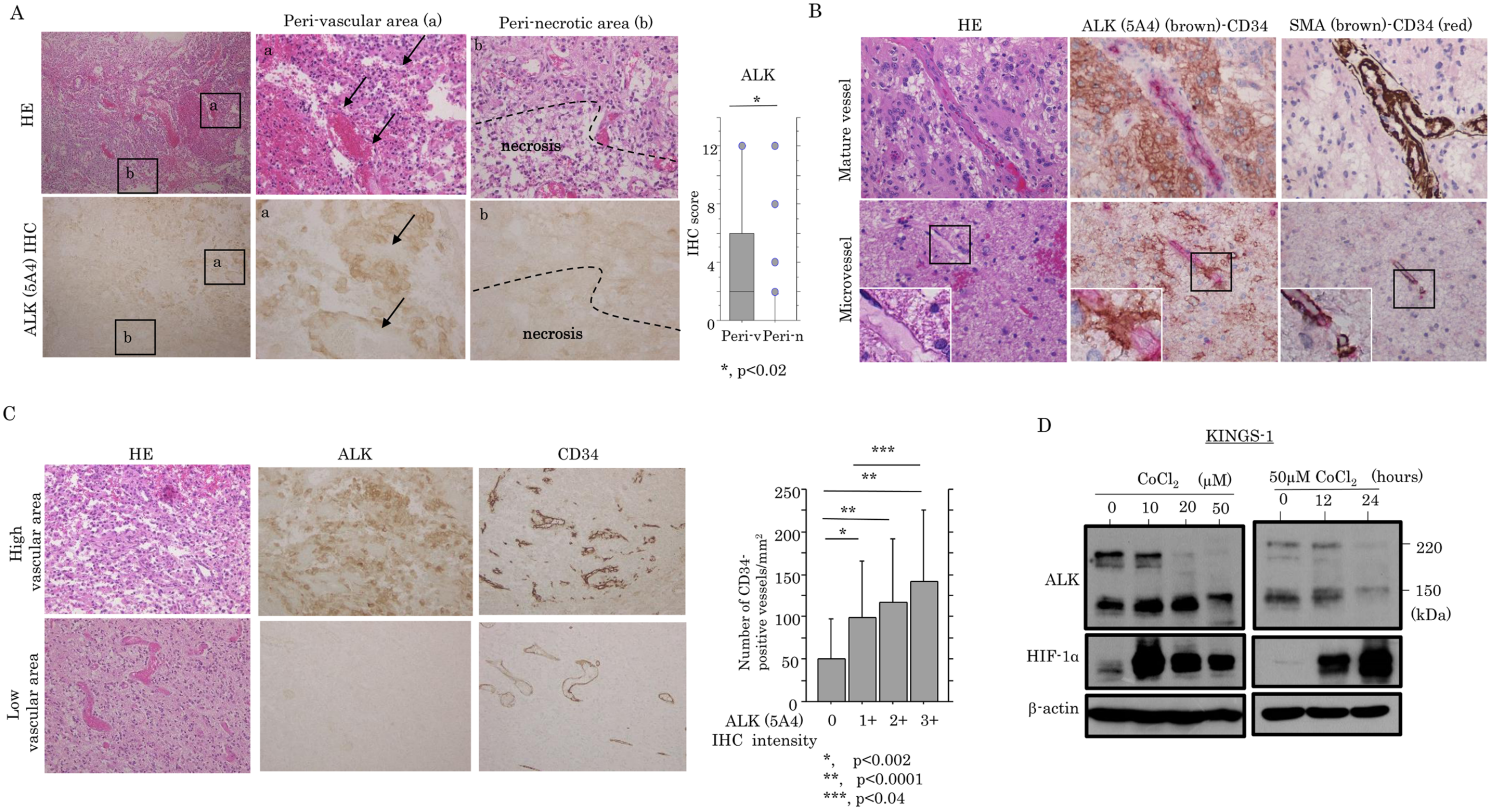
Up-regulation of ALK expression in hypervascular areas of GBMs. (A) Left: staining by hematoxylin and eosin (HE) and IHC for ALK in GBMs. Note the strong cytoplasmic ALK (5A4) positivity in cells around vascular components (a) (indicated by arrows) of GBMs, in contrast to the sporadic staining or absence in tumor cells adjacent to necrotic foci (b) (partitioned by dotted line). Panels (a) and (b) are magnified views of the boxed areas. Original magnification, x100 (left) and x200 (middle and right). Right: IHC score for ALK (5A4) in perivascular (Peri-v) and perinecrotic (Peri-n) areas. (B) Staining by HE (upper and lower left) and double-staining for ALK/CD34 (upper and lower middle) and SMA/CD34 (upper and lower right) in mature vessels (upper) and microvessels (lower) in GBM. Strong ALK (5A4) (brown) positivity is observed in GBM cells surrounding CD34 (red)-positive mature vessels (upper middle), while focal ALK (5A4) immunoreactivity is also evident in CD34-positive microvessels (lower middle). Note the close association between SMA- and CD34-positive cells in both tumor vasculatures (upper and lower left). Insets show magnified views of the boxed areas. Original magnification, x200 and x400 (inset). (C) Left: staining by HE and IHC for ALK and CD34 in GBMs. Note the strong ALK (5A4) immunopositivity in areas with high CD34 immunoreactivity, in contrast to the sporadic staining or absence in the low immunoreactivity lesions. Original magnification, x100. Right: relationship between ALK (5A4) immunointensity and microvascular density as determined by CD34 immunoreactivity in GBMs. The data shown are means±SDs. (D) Western blot analysis of the indicated proteins after CoCl_2_ treatment with the different doses shown for 24 hours (left) and 50 μM CoCl_2_ for the time shown (right) in KINGS-1 cells.

In GBM tissues, strong ALK (5A4) immunoreactivity was frequently observed in perivascular tumor cells, in contrast to the relatively weak expression in those of perinecrotic areas, a difference that was significant based on the ALK (5A4) scores (Fig 2A). Mature vessels, which were characteristically surrounded by mural cells with strong SMA immunoreactivity [28], showed a lack of ALK (5A4) immunoreaction, while a focal ALK (5A4) immunoreactivity was observed in cells expressing either CD34 or SMA in some microvascular components (Fig 2B). ALK (5A4) immunoreaction was also significantly associated with microvascular density (MDV) as determined by CD34 immunopositivity, which is a marker for endothelial cells of blood vessels, but not lymphatics (Fig 2C). To further examine whether ALK expression was affected by the oxygen environment, treatment with CoCl_2_, known to mimic hypoxic conditions [35], was carried out in KINGS-1 cells, which express full-length ALK expression (S6 Fig). As shown in Fig 2D, ALK expression was decreased in dose- and time-dependent manners under hypoxic conditions, in contrast to stabilization of HIF-1α protein.

**Fig 3:**
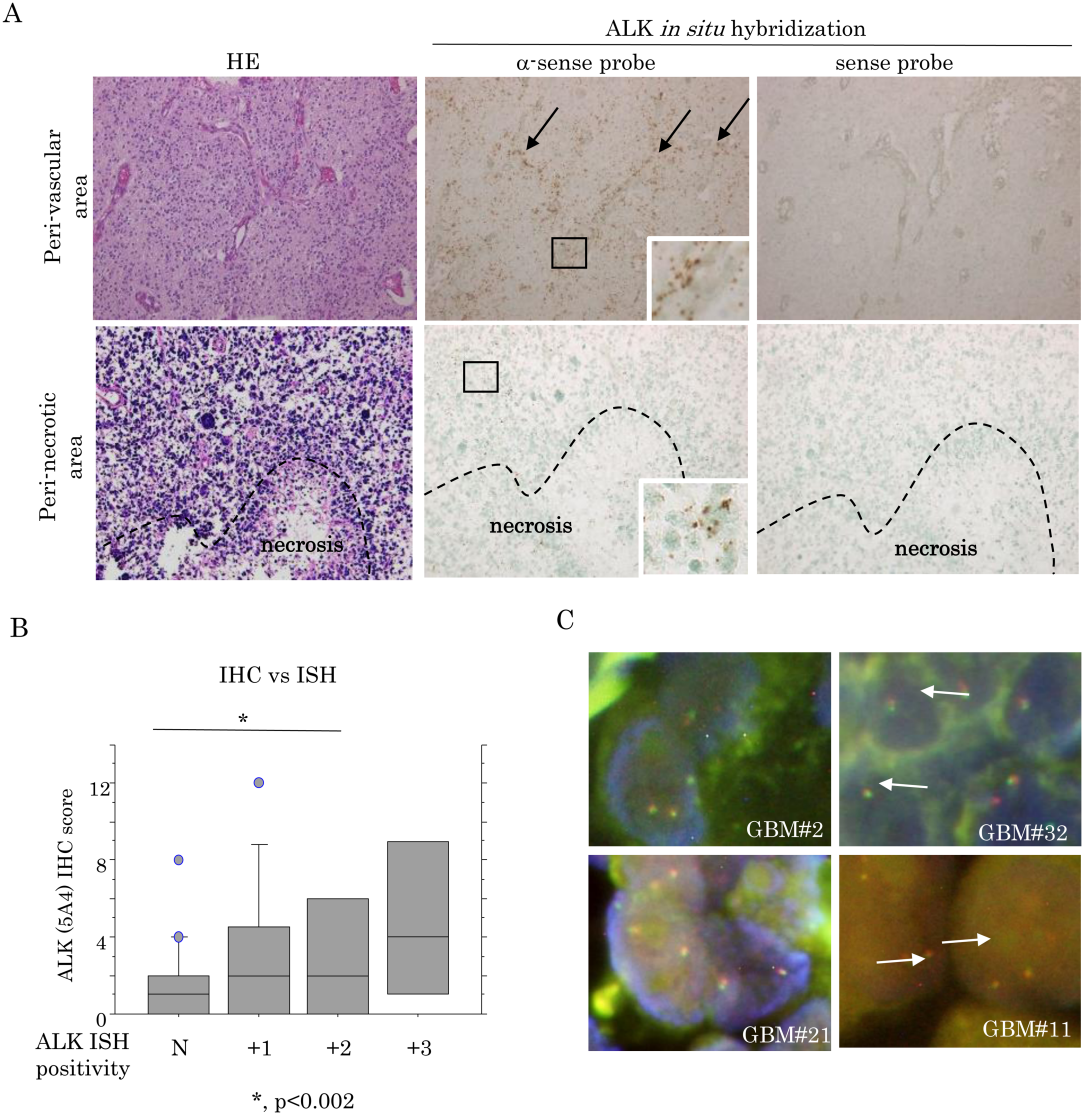
ALK mRNA expression and gene arrangement status in GBMs. (A) Staining by hematoxylin and eosin (HE) and ISH for ALK mRNA. Note the abundant mRNA signals in tumor cells around vascular components (indicated by arrows), in contrast to the weak signals adjacent to necrotic foci (partitioned by dotted line). Insets show magnified views of the boxed areas. Original magnification, x100 and x400 (inset). (B) Relationship of ALK (5A4) expression between the ISH signal positivity and the IHC score in GBMs. (C) FISH analysis of four GMB cases with high ALK expression. The interphase nuclei of these cases indicate absence of ALK rearrangement, as shown by the merged red and green signals (indicated by arrows).

By *in situ* hybridization assay, positive signals for ALK mRNA were detected in 30 (76.9%) of 39 GBM cases, particularly in perivascular lesions (Fig 3A). The ALK ISH positivity tended to be positively associated with the ALK (5A4) scores (Fig 3B). In four GBM cases with strong ALK (5A4) immunoreactivity, the FISH assay revealed no rearrangement or amplification of the *ALK* locus (Fig 3C), and none of the cases had any mutations in exons 20, 23, 24, and 25 of the *ALK* gene (S7 Fig).

**Fig 4:**
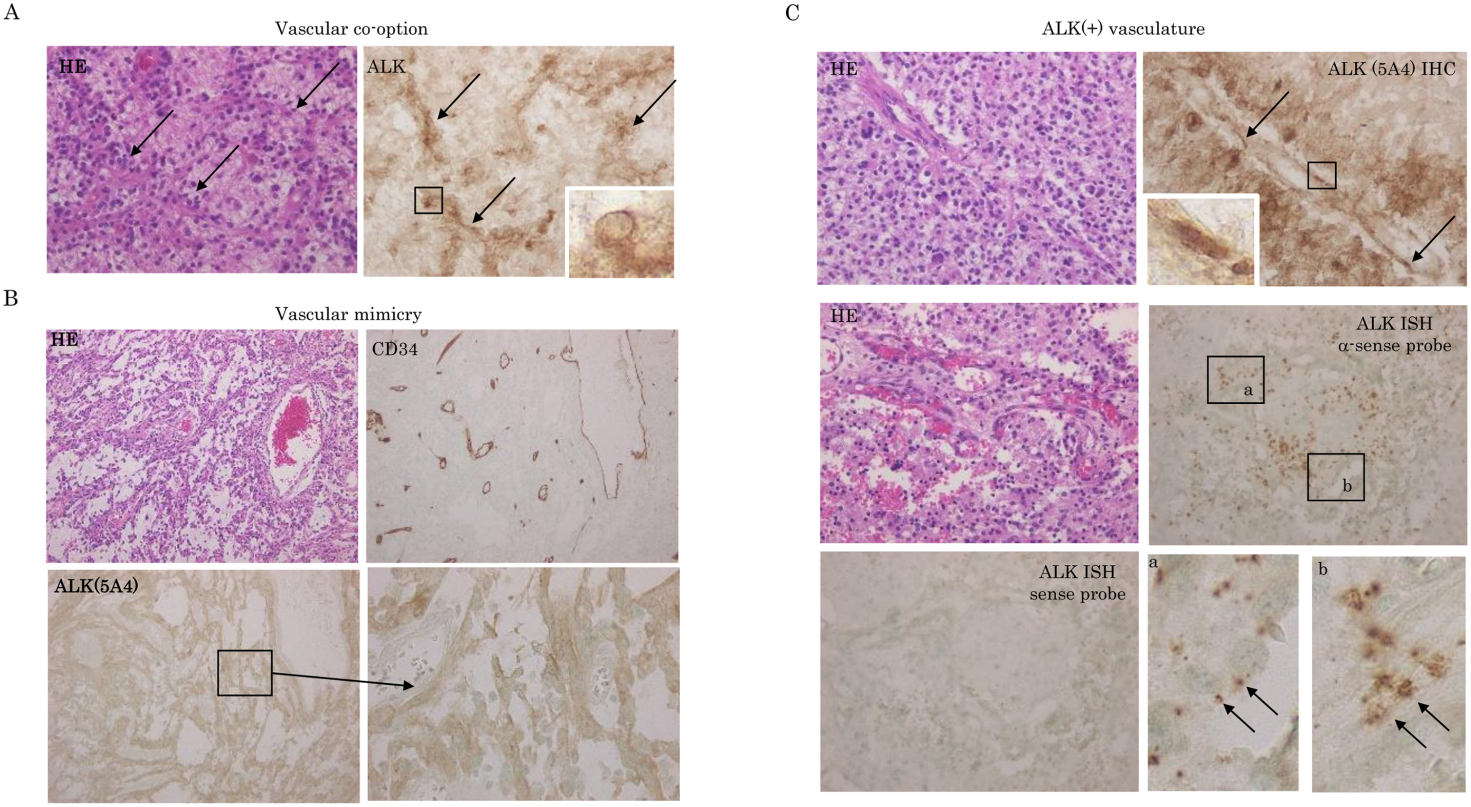
Association between ALK expression and neovascular features in GBMs. (A) Staining by hematoxylin and eosin (HE) and IHC for ALK in vascular co-option. Note the strong ALK (5A4) immunoreactivity in vascular co-option with classic perivascular cuffs (indicated by arrows). Insets show magnified view of the boxed area. Original magnification, x200 and x400 (inset). (B) Staining by HE and IHC for ALK and CD34 in vascular mimicry. Note the diffuse ALK (5A4) immunoreaction and focal CD34 immunoreactivity in the perfused vascular networks containing red blood cells. The lower right panel shows the magnified view of the boxed area in the lower left panel. Original magnification, x100 and x400 (lower right panel). (C) Upper: staining by HE and IHC for ALK. Note the strong ALK (5A4) immunoreactivity in tumor vasculature (indicated by boxes and magnified in the inset), as well as tumor cells around vascular components. Original magnification, x200 and x400 (inset). Middle and lower: staining by HE and ISH for ALK mRNA in GBMs. Note the positive ALK mRNA signals in tumor vasculature which are indicated by boxes in middle right panel and magnified in lower right panels (positive ALK mRNA signal in tumor vasculature are indicated by arrows), as well as tumor cells around vascular components. Original magnification, x200 and x400 (lower left panel).

Strong ALK (5A4) protein expression in vascular co-option components was observed in tumor periphery areas adjacent to non-neoplastic brain tissues (Fig 4A and Table 1). Diffuse immunoreaction was also evident in vascular mimicry channels with CD34-/PAS+ phenotype (Fig 4B, Table 1 and S1 Fig). In addition, ALK mRNA and protein expression were detected in tumor microvasculatures composed of endothelial cells and mural cells (Fig 4C and Table 1).

**Table 1:** Relationship between ALK expression and neovascularization in GBMs.

	N	Vascular co-option n (%)	N	Vascular mimicry n (%)	N	Tumor vasculature n (%)
**Total cases**	50	12 (24)	50	9 (18)	*	*
**ALK (5A4) positivity**	12	8 (67)	9	6 (67)	50	8 (16)

GBM, glioblastoma; N and n, number of cases;

*, not examined

### Association of ALK expression with Stat3/HIF-1α/VEGF-A axis

Based on the above IHC findings, we further examined a possible role of ALK in the promotion of neovascularization by GBM cells. Cell lines stably overexpressing full-length ALK (KS-ALK#4) were established using KS-1 cells which lack endogenous ALK expression (S4 Fig). Two independent cell lines with knockdown of ALK expression by inhibition with ALK-specific shRNA were also established using KINGS-1 cells (KING-shALK#37 and #46).

**Fig 5:**
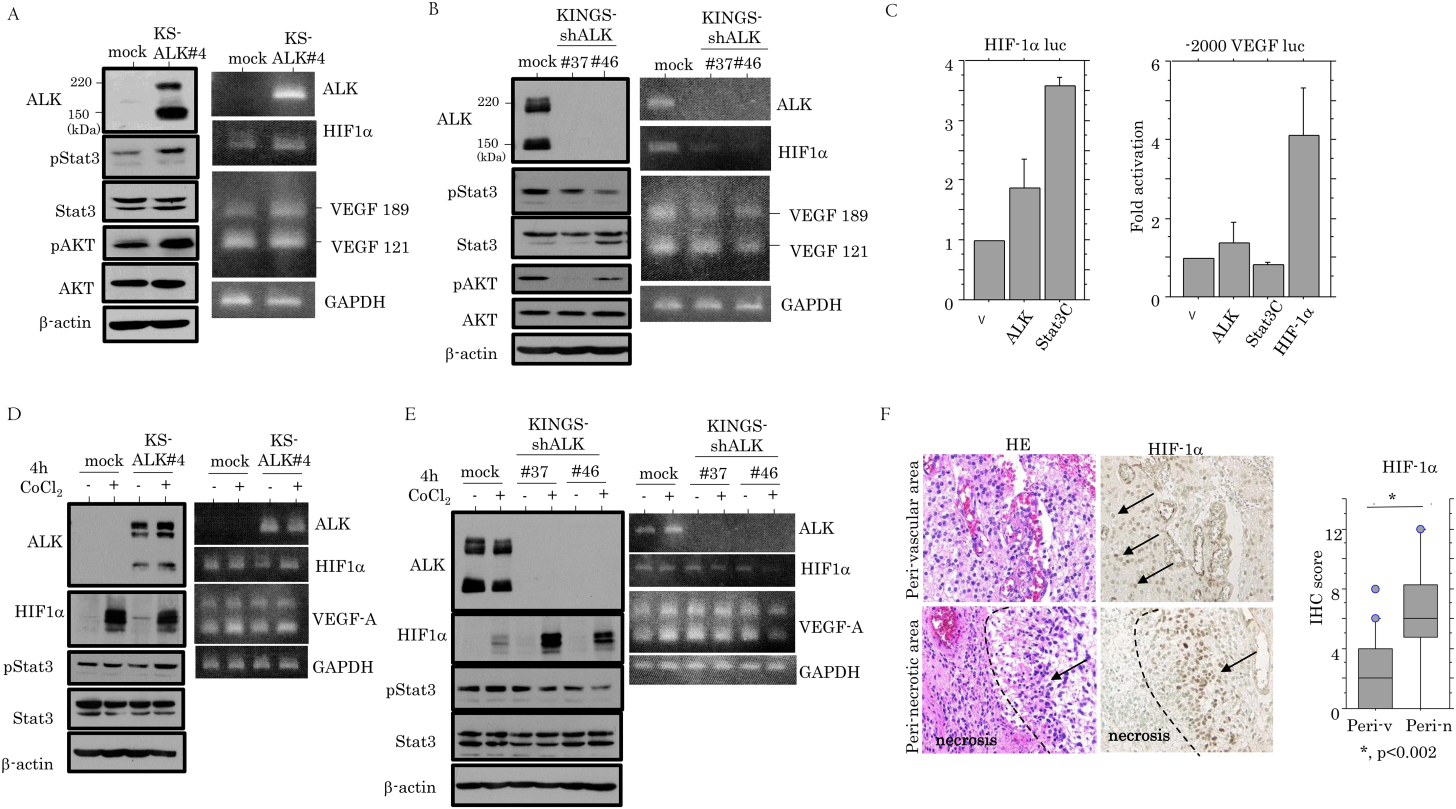
ALK/Stat3/HIF-1α axis in astrocytoma cells. Western blot (left) and RT-PCR (right) analyses for the indicated molecules in (A) KS-ALK#4 cells and (B) KINGS-shALK#37 and #46 cells. (C) KS-1 cells were transfected with HIF-1α (left) and VEGF-A (right) reporter constructs, together with either ALK, Stat3C, or HIF-1α. Relative activity was determined based on arbitrary light units of luciferase activity normalized to pRL-TK activity. The activities of the reporter plus the effector relative to that of the reporter plus empty vector are shown as means±SDs. The experiment was performed in duplicate. v, empty vector. Western blot (left) and RT-PCR (right) analyses for the indicated molecules in (D) KS-ALK#4 cells and (E) KINGS-shALK#37 and #46 cells after CoCl_2_ treatment for 4 hours. (F) Left: staining by hematoxylin and eosin (HE) and IHC for HIF-1α in GBMs. Note the strong HIF-1α immunoreactivity (indicated by arrows) in both perivascular areas and pseudopalisading around necrotic lesion (partitioned by dotted line). Original magnification, x100. Right: IHC score for HIF-1α in perivascular (Peri-v) and perinecrotic (Peri-n) areas of GBMs.

Expression of pStat3 and pAkt proteins, as well as HIF-1α and VEGF-A mRNAs, were increased in the KS-ALK#4 cells as compared to the mock cells (Fig 5A), in contrast to the downregulation of these molecules in the KINGS-shALK cells (Fig 5B). Transient transfection of Stat3C, as well as ALK, induced an increase in *HIF-1*α promoter activity, while overexpression of HIF-1α resulted in enhancement of *VEGF-A* promoter activity (Fig 5C).

Short-term exposure of KS-ALK#4 cells to CoCl_2_ did not affect the expression of these molecules as compared to those in the mock cells (Fig 5D). In contrast, treatment of KINGS-shALK#46 cells with CoCl_2_ resulted in decreased mRNA expression of HIF-1α and VEGF-A (Fig 5E). In GBM tissues, although HIF-1α immunoreactivity was detected in not only perinecrotic lesions, but also perivascular areas within tumor tissues (Fig 5F), the HIF-1α score was not associated with ALK and pStat3 scores in GBMs (data not shown).

### ALK enhances cell proliferation by up-regulation of pAkt and pStat3

**Fig 6:**
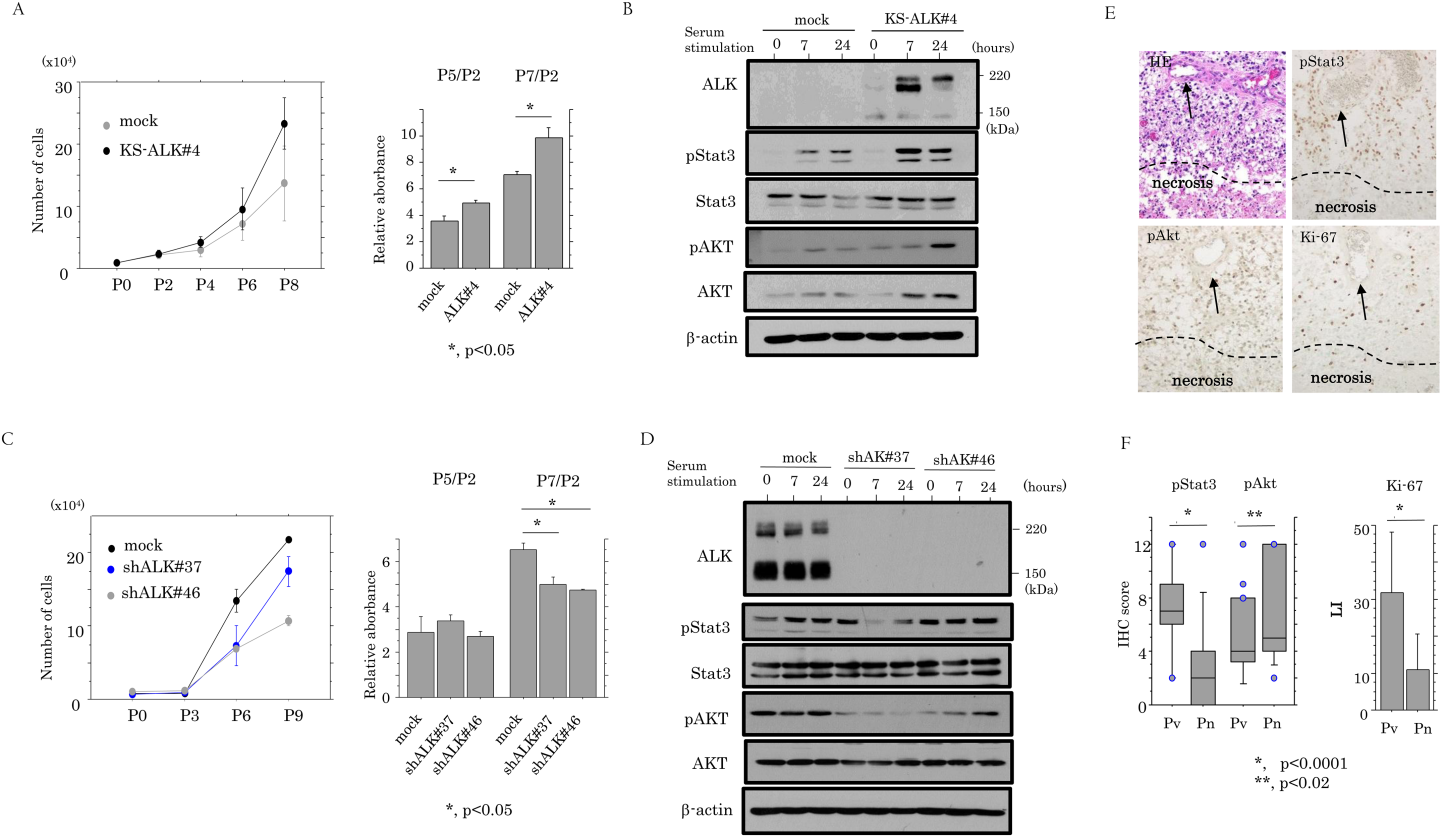
Association between ALK expression and cell proliferation in astrocytoma cells. (A) Left: KS-ALK#4 cells and mock cells were seeded at low density. The cell numbers are presented as means±SDs. P0, P2, P4, P6, and P8 indicate 0, 2, 4, 6, and 8 days after cell passage, respectively. Right: Cell Counting Kit-8 (CCK-8) assay for cell proliferation. Cells were seeded at 1x10^3^ cells in 96-well plates. Viable cell numbers were quantitated. Relative absorbance values (P5 or P7 relative to P2) are presented as means±SDs. P2, P5, and P7 indicate 2, 5, and 7 days after cell passage, respectively. This experiment was performed in triplicate using independent samples. (B) Western blot analysis for the indicated proteins in ALK#4 and mock cells after serum stimulation for the times shown. (C) Left: KINGS-shALK#37, #46 cells, and mock cells were seeded at low density. The cell numbers are presented as means±SDs. P0, P3, P6, and P9 indicate 0, 3, 6, and 9 days after cell passage, respectively. Right: CCK-8 assay for cell proliferation to quantitate viable cell numbers as mentioned above. (E) Staining by hematoxylin and eosin (HE) and IHC for pStat3, pAkt, and Ki-67 in GBMs. Note the strong immunoreactivity for these molecules in perivascular lesions (vessels are indicated by arrows), in contrast to the weak immunoreaction in perinecrotic areas (necrotic lesion is partitioned by dotted line). Original magnification, x100. (F) IHC scores for pStat3 and pAkt and Ki-67 labeling indices in perivascular (Pv) and perinecrotic (Pn) lesions.

Given that continuous activation of ALK-related signals leads to the persistent modulation of target genes, which govern key cell functions such as proliferation [36], we examined whether ALK expression was associated with changes in cell kinetics. The KS-ALK#4 stable cells demonstrated a tendency towards a higher proliferation rate, particularly in the exponential growth phase, as compared to the mock cells (Fig 6A). When the stable cells were rendered quiescent by serum starvation and were subsequently stimulated with serum, expression levels of pStat3 and pAkt were substantially increased relative to the mock cells at 6 and 24 h after release of the cell cycle (Fig 6B), in contrast to the KINGS-shALK cells that showed a lower proliferation rate (Fig 6C) and relatively minor changes in pAkt expression (Fig 6D). In GBM tissues, pStat3 score and Ki-67 LIs, but not pAkt score, were significantly higher in perivascular lesions as compared to perinecrotic foci (Fig 6E and 6F). As shown in Table 2, ALK (5A4) score was positively correlated with pStat3 score and Ki-67 LIs, while pAkt score was not associated with any of these markers.

**Table 2:** Correlations among IHC markers investigated in GBM cases.

	**ALK (5A4) score** ***ρ* (p)**	**N-myc score** ***ρ* (p)**	**c-myc score** ***ρ* (p)**	**pAkt score** ***ρ* (p)**	**pStat3 score** ***ρ* (p)**
				*	*
**N-myc score**	0.44	*	*		
	<0.0001				
**c-myc score**	0.34	0.21	*	*	*
	0.003	0.06			
**pAkt score**	0.15	0.2	0.35	*	*
	0.2	0.11	0.004		
**pStat3 score**	0.44	0.55	0.23	-0.08	*
	0.0001	<0.0001	0.04	0.46	
**Ki-67 LI**	0.4	0.11	0.17	-0.01	0.37
	0.0004	0.33	0.13	0.91	0.001

*ρ*, Spearman's correlation coefficient; IHC, immunohistochemistry; LI, labeling index

*, not examined

### Activation of ALK promoter by Sox4 and N-myc

**Fig 7:**
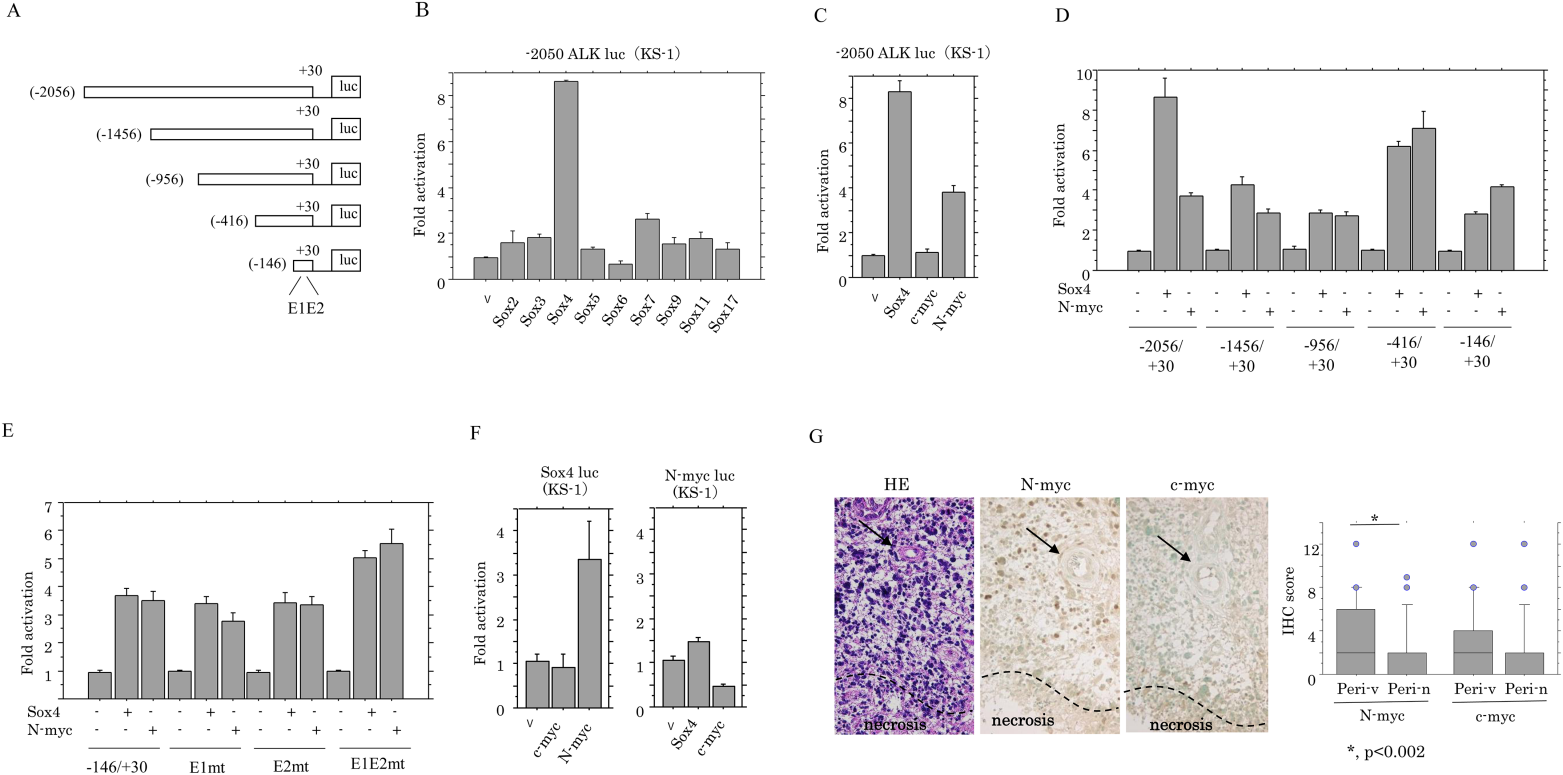
Relationship of ALK expression with Sox4 and N-myc in astrocytomas. (A) Various *ALK* promoter constructs used in this study. (B) KS-1 cells were transfected with *ALK* promoter constructs, together with the indicated *Sox* genes. Relative activity was determined based on arbitrary light units of luciferase activity normalized to pRL-TK activity. The activities of the reporter plus the effector relative to that of the reporter plus empty vector are shown as means±SDs. The experiment was performed in duplicate. (C) KS-1 cells were transfected with *ALK* promoter constructs, together with either Sox4, c-myc, or N-myc. (D) Various promoter constructs were used for evaluating transcriptional regulation of the *ALK* promoter by either Sox4 or N-myc. (E) The shortest *ALK* promoter constructs containing mutations in two putative E-boxes (E1 and E2), along with either Sox4 or N-myc, were transfected into KS-1 cells. (F) The Sox4 (left) and the N-myc (right) promoter constructs, along with either c-myc, N-myc, or Sox4, were transfected into KS-1 cells. (G) Left: staining by hematoxylin and eosin (HE) and IHC for N-myc and c-myc in GBMs. Note the strong immunoreactivity for N-myc, but not c-myc, in tumor cells around vessel (indicated by arrows) but not perinecrotic area (necrotic lesion is partitioned by dotted line). Right: IHC scores for N-myc and c-myc in perivascular (Peri-v) and perinecrotic (Peri-n) lesions.

Since some *Sox* genes are essential for development of general neuronal properties [37], we first examined the association between several Sox factors and ALK expression. Transient transfection of the longest *ALK* promoter constructs (Fig 7A), along with nine Sox factors, revealed that only Sox4 resulted in increased activity of the *ALK* promoter (Fig 7B). A similar effect was also observed by transfection of N-myc, but not c-myc (Fig 7C). Using a series of 5’-truncated promoter constructs (Fig 7A), we found that deletion from -2056 to -146 bp had little effect on induction of the *ALK* promoter activity by both Sox4 and N-myc, and the shortest construct (-146/+30 bp), which lacks putative Sox-binding sites, still preserved the responsiveness to both Sox4 and N-myc activation (Fig 7D). Although four nucleotide alterations in E-boxes, which are binding sites for N-myc, were introduced in the shortest ALK construct, changes in *ALK* promoter activity by Sox4 and N-myc stimulation were relatively minor (Fig 7E). Finally, transfection of N-myc, but not c-myc, also resulted in increased *Sox4* promoter activity, while *N-myc* promoter was not affected by Sox4 and c-myc expression (Fig 7F). In GBM tissues, N-myc, but not c-myc, score was significantly higher in perivascular lesions than perinecrotic areas, and was positively correlated with ALK (5A4) score (Fig 7G and Table 2).

## Discussion

The present study clearly provided evidence that full-length ALK without any chromosomal rearrangements or gene mutations was frequently overexpressed in astrocytomas, particularly in GBMs. The OS and PFS of patients with ALK-immunopositive tumors were significantly lower than those showing a lack of ALK expression. Further, our results demonstrated that ALK signaling involves the activities of N-myc, Sox4, Sta3, and Akt, which together promote increases in tumor neovascularization under non-hypoxic conditions and cell proliferation.

Both N-myc and c-myc have been demonstrated to be capable of inducing the proximal promoter activity of the *ALK* gene through direct interaction with the E-boxes in neuroblastoma cells [38]. Our results showed, however, that only N-myc could enhance *ALK* promoter activity in GBM cells, independent of the presence of E-boxes. This is in line with the IHC findings showing that N-myc, but not c-myc, score was significantly higher in perivascular lesions than in perinecrotic areas and was positively correlated with ALK score in GBM tissues. In addition to N-myc, Sox4, which directly activates early genes that endow cells with general neuronal properties [37], was also able to enhance the transcription of *ALK* gene, despite a lack of Sox4-binding sites in the proximal promoter regions. Given the evidence that Sox proteins generally exhibit their gene regulatory functions only by forming complexes with partner transcription factors [39], it is possible that Sox4, as well as N-myc, may require some cell type-specific factors to regulate ALK transcription. In addition, activation of *Sox4* promoter by N-myc indicates that N-myc may serve as a critical upstream factor in activation of the ALK signaling cascade in GBMs.

Several lines of evidence from our present data support the conclusion that ALK signaling may contribute to the non-hypoxia-driven mechanism of neovascularization in GBMs. First, ALK expression was significantly higher in tumor cells in hypervascular lesions as compared to those adjacent to necrotic foci in GBMs, and was positively correlated to the microvascular density as determined by CD34 expression. Moreover, cells under hypoxic conditions induced by CoCl_2_ treatment showed a decrease in ALK expression, suggesting that non-hypoxic tumor microenvironment may be essential for maintaining ALK signaling activity. This conclusion is supported by a report showing that N-myc expression was distinctly down-regulated in hypoxic neuroblastoma cells with N-myc amplification [40]. Second, ALK-positive cells appeared to be closely linked with neovascularization features including vascular co-option and vascular mimicry in GBMs. It has been proposed that vascular mimicry represents an incomplete trans-differentiation of cancer stem cells toward an endothelial phenotype [41]. Given that an overlap is evident from a recent report showing both vascular mimicry and trans-differentiation [4], it is likely that ALK expression may be associated with GBM-endothelial cell trans-differentiation. Although we were unable to determine the phenotype of cells expressing ALK in tumor microvasculatures due to a close association between endothelial cells and mural cells in the components, it has been reported that the most intense ALK staining was found in the mural cells of tumor vessels in human gliomas [24]. Third, overexpression of ALK induced an enhancement of the HIF-1α/VEGF-A axis through activation of Stat3, while knockdown of ALK resulted in decreased expression of these molecules. These findings were in line with a report showing Stat3-mediated activation of *HIF-1*α gene transcription by nucleophosmin/ALK in ALK-positive T-cell lymphoma [25].

Activation of Stat3 and Akt by oncogenic tyrosine kinases has been reported to regulate the expression of genes that are essential for cell growth and survival [22]. Our results demonstrated that changes in ALK expression were also involved in modulation of proliferation of GBM cells, leading to alterations in the expression of Stat3 and Akt. Our IHC findings, however, revealed that while ALK score was positively correlated with pStat3 score and Ki-67 LIs in GBM tissues, pAkt score showed lack of direct associations with ALK and pStat3 scores and Ki-67 LIs. It is possible that other oncogenic signaling pathways that active pAkt may exist in parallel or have crosstalk with ALK signal transduction in GBMs. In fact, overexpression of pAkt in response to hypoxic effects was reported to be observed in pseudopalisading area around necrotic foci in GBMs [35].

Our study was limited by the higher proportion of GBM patients showing strong ALK expression which may have biased the OS and PFS. In addition, the lack of effect of ALK on the prognostic values of *IDH1* gene alterations may be attributed to the small number of patients in our prospective cohort and a few cases of secondary GBM among patients with ALK expression. Further, although ALK mRNA expression as detected by ISH was significantly associated with the immunoreactivity, some cases also showed weak positive ISH signals despite a negative immunoreaction, particularly in perinecrotic areas. This may be due to the difference in detection sensitivity between the two assays. In addition, post-transcriptional and post-translational modifications of ALK may have also occurred in certain cell types, since discrepant results between ALK transcript and protein expression have also been demonstrated in some melanoma and lung cancer cell lines [42].

## Conclusions

**Fig 8:**
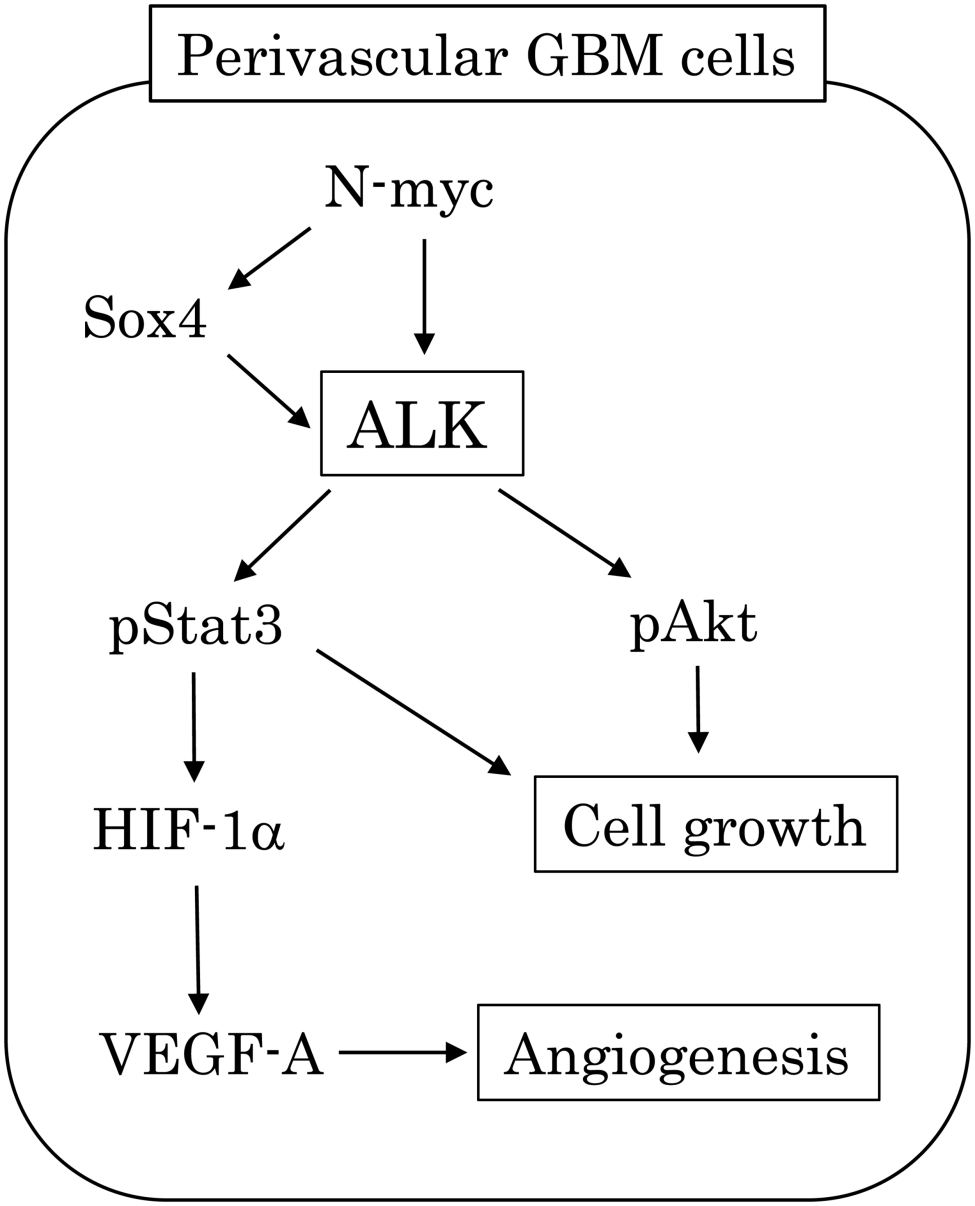
Schematic representation of ALK signal networks in modulation of neovascularization and cell proliferation in perivascular GBM cells.

Together, our findings suggest that ALK signaling has novel functional roles in GBMs, particularly in perivascular lesions (Fig 8). Overexpression of N-myc, along with Sox4, induces transcriptional up-regulation of the *ALK* gene, which in turn triggers activation of downstream transduction cascades containing Stat3, Akt, HIF-1α, and VEGF-A, resulting in an increase in cell proliferation and enhancement of neovascularization. Given that N-myc acts as a key effector of PI3K-meditated VEGF expression in neuroblastomas [43], it appears that positive feedback loops among N-myc, ALK, and Akt pathways may exist in perivascular GBM cells, leading to deregulation of VEGF expression. Thus, constitutive ALK activation confers multiple advantages to tumor cells that are essential for successful malignant progression. Additional *in vivo* model assays are clearly warranted to further validate our findings.

## Supporting information

S1 Fig Overexpression of ALK in lung carcinoma tissue (case 170718) with gene rearrangement. Upper left and right and lower right: staining by hematoxylin and eosin (HE) and IHC for ALK. Cytoplasmic ALK immunoreactivity is detected by two independent anti-ALK antibodies including clones 5A4 (upper right) and D5F3 (lower right). Note the strong immunoreactivity in the former as compared to that in the latter. Lower left and middle: FISH analysis of *ALK* gene using ProbeCheck ALK Positive Control Slides (lower left: positive control) and lung carcinoma tissue (lower middle: case 170718). Note the two red signals (indicated by arrows) which indicate the presence of *ALK* gene rearrangements.
(TIF)

S2 Fig CD34/PAS double-staining in vascular mimicry channels in serial sections of GBM. Staining by hematoxyline and eosin (HE) (upper left) and CD34/PAS double-staining (upper right) in vascular mimicry channels. Both CD34-/PAS+ (a; indicated by long arrows) and CD34+/PAS+ (a; indicated by short arrow) vessels are demonstrated around vascular mimicry channels with PAS-positive deposition on luminal surface lined by tumor cells (b; indicated by arrows). Note the red blood cells (c; indicated by arrows) in the vascular mimicry channels. Insets (a,b,c) show magnified views of the boxed areas in the upper panels. Original magnification, x40 and x400 (inset).
(TIF)

S3 Fig ALK expression detected by the two independent antibodies in GBMs. Staining by hematoxylin and eosin (HE) and IHC for ALK using two independent antibodies including clones 5A4 and D5F3. Immunoreaction with both antibodies is observed in perivascular GBM cells (indicated by arrows). Note the relatively weak immunoreactivity with clone D5F3 (right) as compared to that of clone 5A4 (middle). Original magnification, x100.
(TIF)

S4 Fig Staining by hematoxylin and eosin (HE) and IHC for ALK in normal brain. Note the weak immunoreactivity for ALK (5A4) in nerve cell (indicated by long arrow), in contrast to the lack of immunoreactivity in glia cells (indicated by short arrows). Original magnification, x400.
(TIF)

S5 Fig IDH1 abnormality in astrocytomas. (A) IHC and sequence analysis of *IDH1* gene in grade II astrocytoma. Note the cytoplasmic IDH1 staining (middle; indicated by arrows) and heterozygous mutation (R132H) of *IDH1* gene (right). (B) Relationship of *IDH1* gene status with overall survival and progression-free survival in all grades of astrocytomas. n, number of cases.
(TIF)

S6 Fig Endogenous ALK expression in three astrocytoma cell lines. RT-PCR (left) and western blot assay (right). Note the ALK mRNA and protein expression in KINGS-1 cells, in contrast to the lack of expression in No.10 and KS-1 cells. Hec251 cells stably overexpressing ALK (H251-ALK) were used as a positive control for ALK expression.
(TIF)

S7 Fig Mutation analysis of the *ALK* gene. (A) Staining by hematoxylin and eosin (HE) and IHC for ALK (5A4) in GBM#33 case. (B) Mutation analysis of exons 20, 23, 24, and 25 of *ALK* gene in GBM#33 case. Note the lack of mutations in the four exons.
(TIF)

S1 Table Correlation of IDH 1 between protein and gene status in astrocytomas. (DOCX)

S2 Table Alteration in IDH 1 status in astrocytomas. (DOCX)
