# Supplementary figures and images for: ALK signaling cascade confers multiple advantages to glioblastoma cells through neovascularization and cell proliferation
Source: PLoS One. 2017 Aug 24;12(8):e0183516. doi: 10.1371/journal.pone.0183516 (PMC5570309; doi:10.1371/journal.pone.0183516)

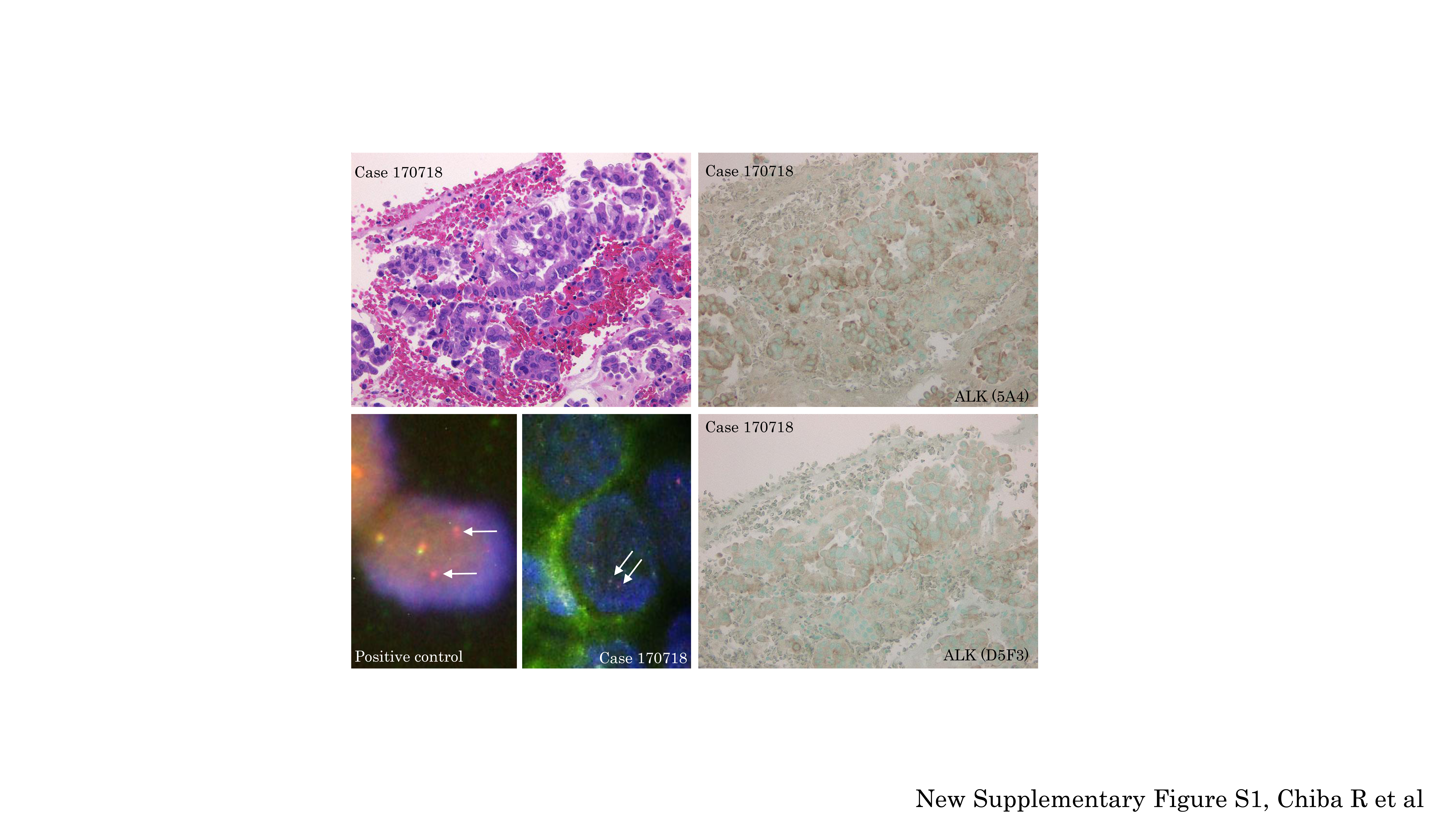

Supplement: S1 Fig — Upper left and right and lower right: staining by hematoxylin and eosin (HE) and IHC for ALK. Cytoplasmic ALK immunoreactivity is detected by two independent anti-ALK antibodies including clones 5A4 (upper right) and D5F3 (lower right). Note the strong immunoreactivity in the former as compared to that in the latter. Lower left and middle: FISH analysis of ALK gene using ProbeCheck ALK Positive Control Slides (lower left: positive control) and lung carcinoma tissue (lower middle: case 170718). Note the two red signals (indicated by arrows) which indicate the presence of ALK gene rearrangements. (TIF) [file pone.0183516.s001.tif]

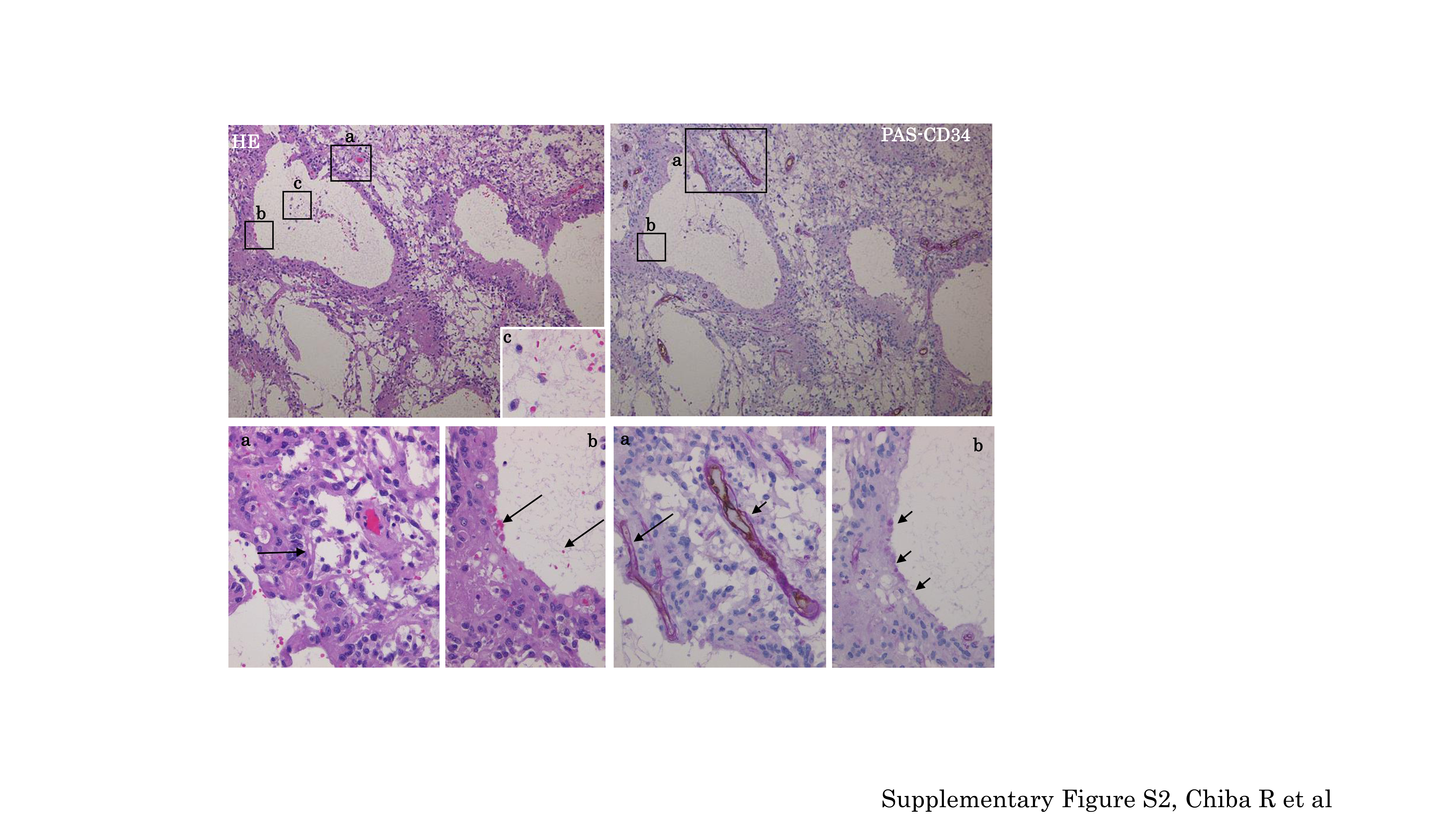

Supplement: S2 Fig — Staining by hematoxyline and eosin (HE) (upper left) and CD34/PAS double-staining (upper right) in vascular mimicry channels. Both CD34-/PAS+ (a; indicated by long arrows) and CD34+/PAS+ (a; indicated by short arrow) vessels are demonstrated around vascular mimicry channels with PAS-positive deposition on luminal surface lined by tumor cells (b; indicated by arrows). Note the red blood cells (c; indicated by arrows) in the vascular mimicry channels. Insets (a,b,c) show magnified views of the boxed areas in the upper panels. Original magnification, x40 and x400 (inset). (TIF) [file pone.0183516.s002.tif]

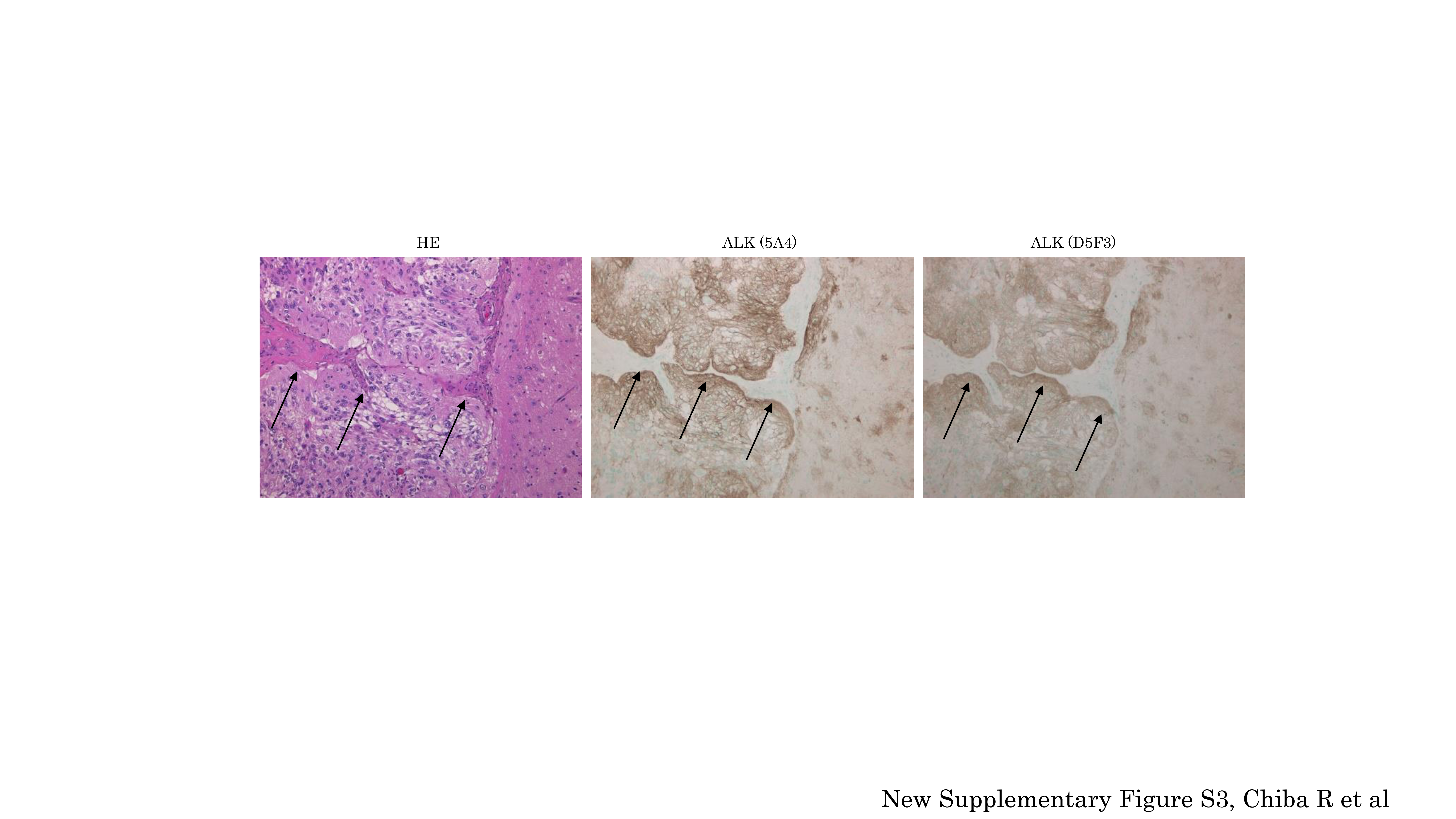

Supplement: S3 Fig — Staining by hematoxylin and eosin (HE) and IHC for ALK using two independent antibodies including clones 5A4 and D5F3. Immunoreaction with both antibodies is observed in perivascular GBM cells (indicated by arrows). Note the relatively weak immunoreactivity with clone D5F3 (right) as compared to that of clone 5A4 (middle). Original magnification, x100. (TIF) [file pone.0183516.s003.tif]

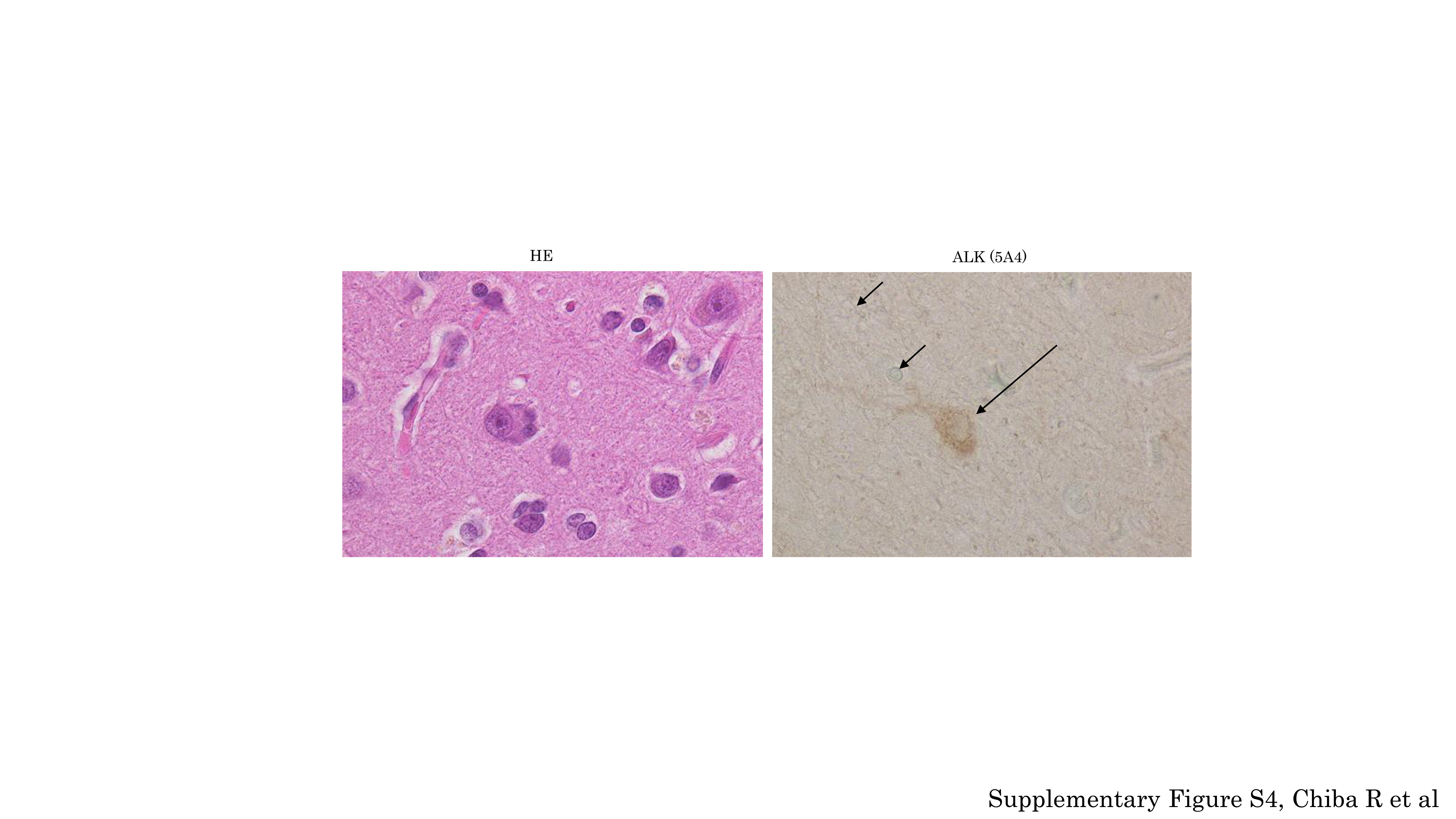

Supplement: S4 Fig — Note the weak immunoreactivity for ALK (5A4) in nerve cell (indicated by long arrow), in contrast to the lack of immunoreactivity in glia cells (indicated by short arrows). Original magnification, x400. (TIF) [file pone.0183516.s004.tif]

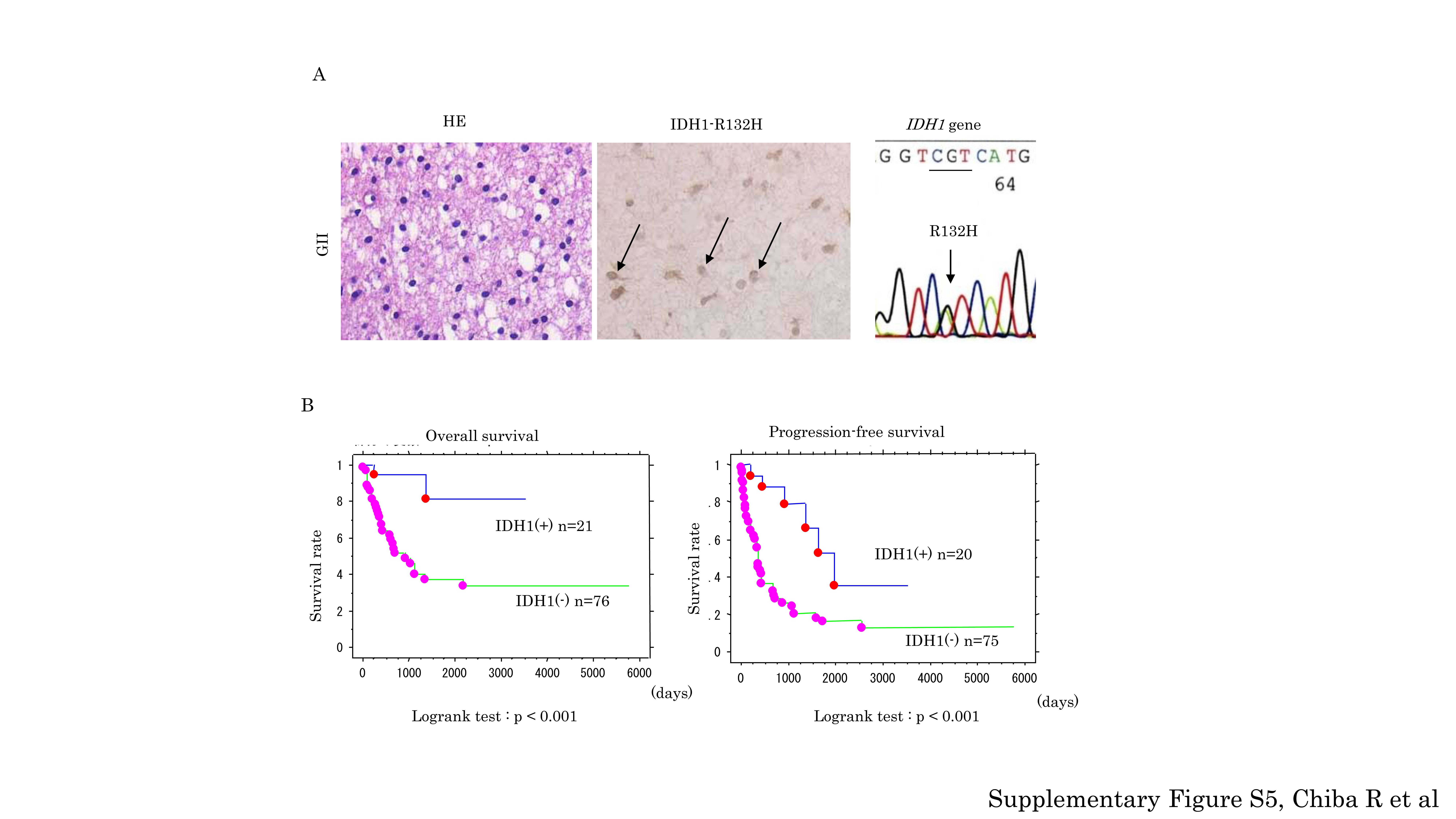

Supplement: S5 Fig — (A) IHC and sequence analysis of IDH1 gene in grade II astrocytoma. Note the cytoplasmic IDH1 staining (middle; indicated by arrows) and heterozygous mutation (R132H) of IDH1 gene (right). (B) Relationship of IDH1 gene status with overall survival and progression-free survival in all grades of astrocytomas. n, number of cases. (TIF) [file pone.0183516.s005.tif]

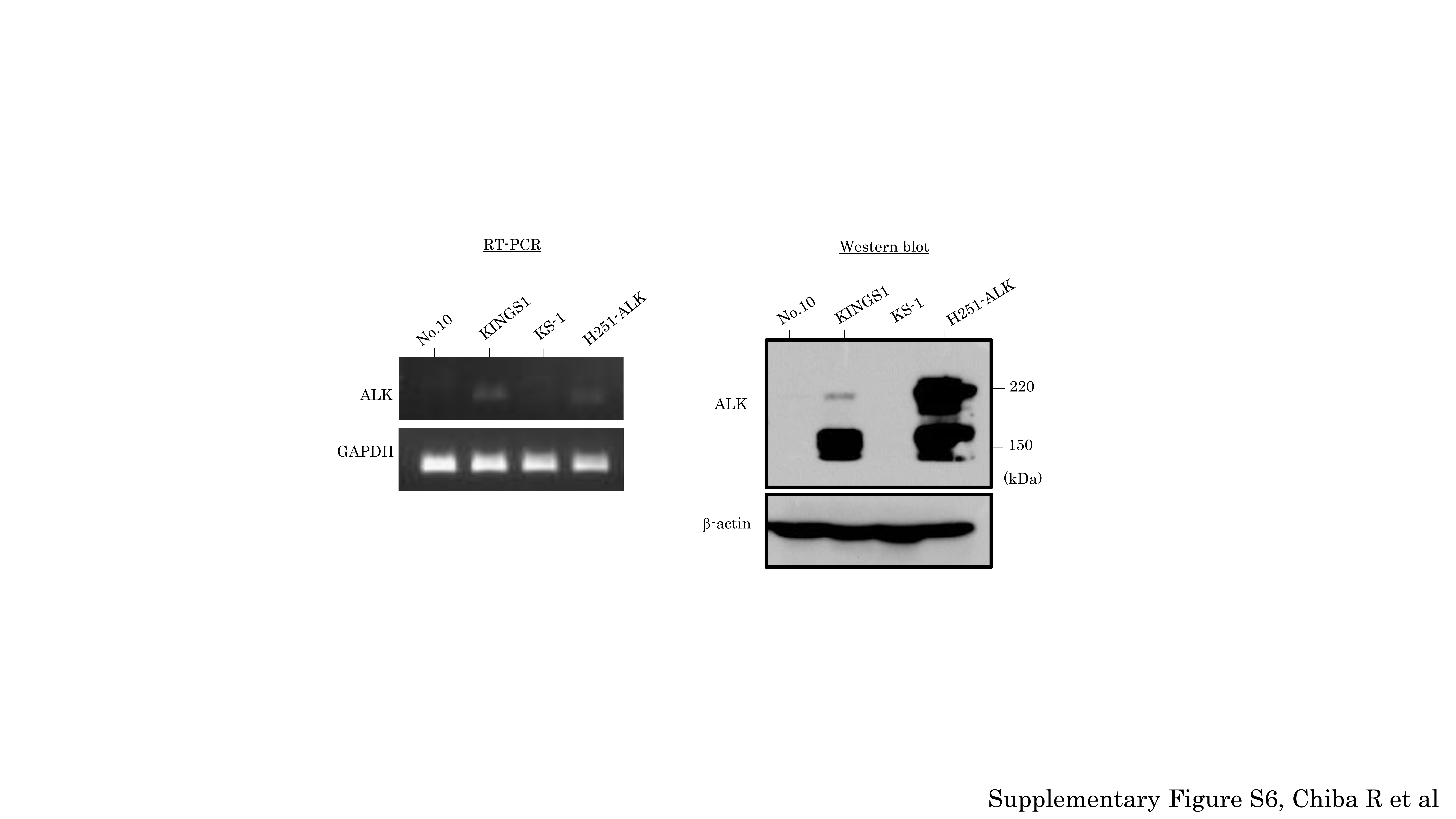

Supplement: S6 Fig — RT-PCR (left) and western blot assay (right). Note the ALK mRNA and protein expression in KINGS-1 cells, in contrast to the lack of expression in No.10 and KS-1 cells. Hec251 cells stably overexpressing ALK (H251-ALK) were used as a positive control for ALK expression. (TIF) [file pone.0183516.s006.tif]

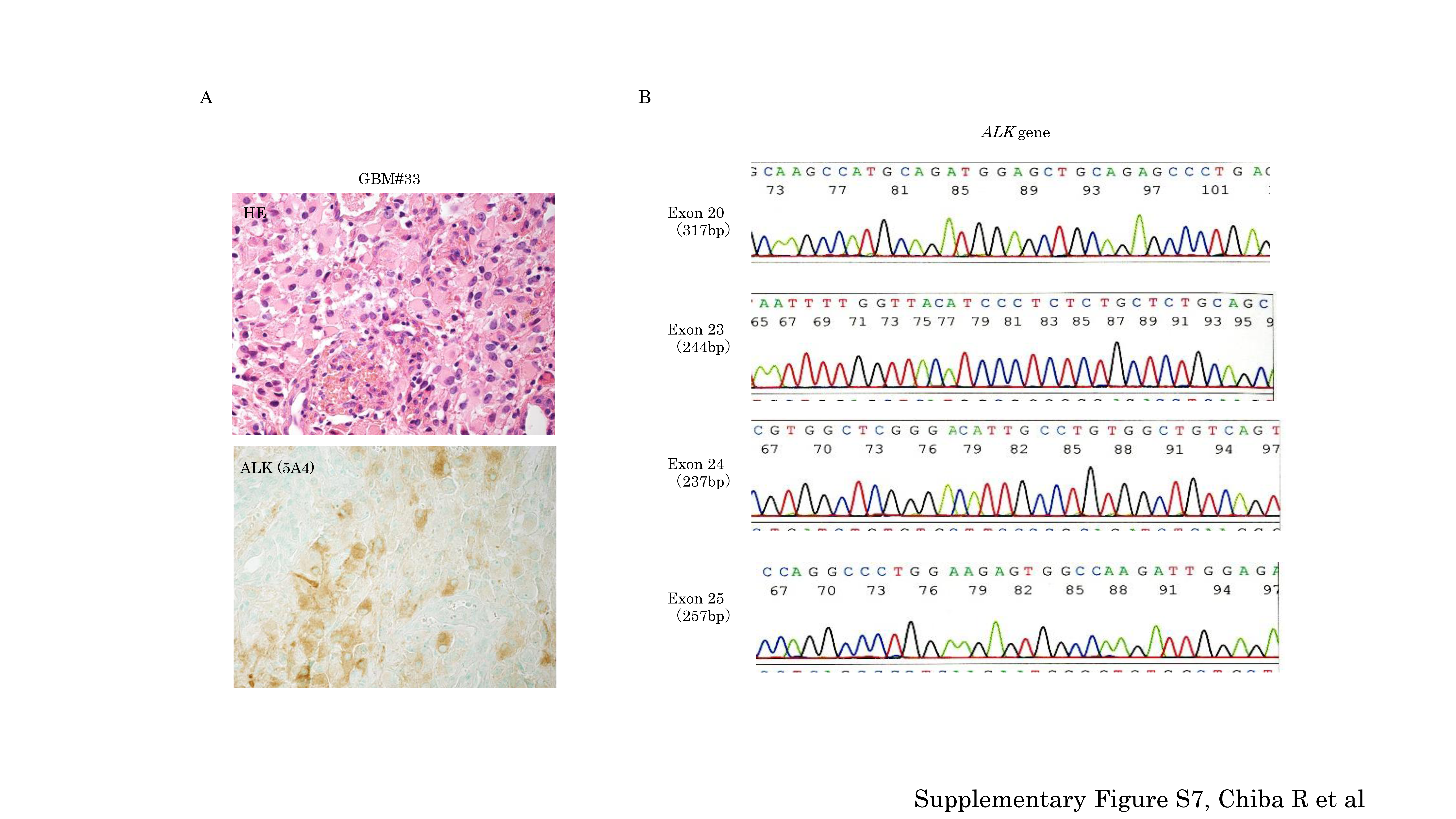

Supplement: S7 Fig — (A) Staining by hematoxylin and eosin (HE) and IHC for ALK (5A4) in GBM#33 case. (B) Mutation analysis of exons 20, 23, 24, and 25 of ALK gene in GBM#33 case. Note the lack of mutations in the four exons. (TIF) [file pone.0183516.s007.tif]
