# Supplementary material for: ALK signaling cascade confers multiple advantages to glioblastoma cells through neovascularization and cell proliferation
Source: PLoS One. 2017 Aug 24;12(8):e0183516. doi: 10.1371/journal.pone.0183516 (PMC5570309; doi:10.1371/journal.pone.0183516)
Supplement: S1 Table — (DOCX) [file pone.0183516.s008.docx]

| **S1 Table. Alteration in isocitrate dehydrogenase 1 status in astrocytomas** | | | | | | | |  |  |
| --- | --- | --- | --- | --- | --- | --- | --- | --- | --- |
|  |  |  |  |  |  |  |  |  |  |
|  |  |  |  |  |  |  |  |  |  |
|  |  | **Protein status (IHC)** | |  |  | **Gene status (sequence)** | |  |  |
|  |  | **Positive** | **Negative** |  |  | **Wild** | **Mutant** |  |  |
|  | N | **n (%)** | **n (%)** |  | **p-value** | **n (%)** | **n (%)** |  | **p-value** |
|  |  |  |  |  |  |  |  |  |  |
| **Grade II** | 34 | 22 (64.7) | 12 (35.3) |  |  | 12 (35.3) | 22 (64.7) |  |  |
|  |  |  |  |  |  |  |  |  |  |
| **Grade III** | 15 | 7 (46.7) | 8 (53.3) |  | <0.0001 | 5 (33.3) | 10 (66.7) |  | 0.005 |
|  |  |  |  |  |  |  |  |  |  |
| **Grade IV** | 50 | 6 (12) | 44 (88) |  |  | 4 (8) | 46 (92) |  |  |
|  |  |  |  |  |  |  |  |  |  |
|  |  |  |  |  |  |  |  |  |  |
| IHC, immunohistochemistry; N and n, number of cases | | | | | |  |  |  |  |
