# Supplementary material for: ALK signaling cascade confers multiple advantages to glioblastoma cells through neovascularization and cell proliferation
Source: PLoS One. 2017 Aug 24;12(8):e0183516. doi: 10.1371/journal.pone.0183516 (PMC5570309; doi:10.1371/journal.pone.0183516)
Supplement: S2 Table — (DOCX) [file pone.0183516.s009.docx]

| **S2 Table. Correlation of isocitrate dehydrogenase between** | | | | |
| --- | --- | --- | --- | --- |
| **between protein and gene status in astrocytomas** | | | |  |
|  |  |  |  |  |
|  |  | **Protein status (IHC)** | |  |
|  |  | **Positive** | **Negative** | **p-value** |
|  |  |  |  |  |
| **Gene status** | **Wild** | 20 | 1 |  |
| **(sequence)** |  |  |  | <0.0001 |
|  | **Mutant** | 15 | 63 |  |
|  |  |  |  |  |
|  |  |  |  |  |
| IHC, immunohistochemistry | |  |  |  |
